# Impact of metastasectomy on survival in patients with oligometastatic stage 4a lung cancer: a retrospective analysis

**DOI:** 10.1007/s13304-025-02120-5

**Published:** 2025-02-06

**Authors:** Ahmet Ulusan, Bekir Elma, Hilal Zehra Kumbasar Danaci, Maruf Sanli, Ahmet Feridun Isik

**Affiliations:** Department of Thoracic Surgery, Faculty of Medicine, Gaziantep University, 27310 Gaziantep, Turkey

**Keywords:** Lung cancer, Oligometastasis, Solitary metastasis, Metastasectomy, Survival

## Abstract

The aim of our study is to evaluate the impact of metastasectomy on survival in patients with oligometastatic stage 4 lung cancer. Fifty-nine oligometastatic lung cancer cases operated on in our clinic between January 2015 and January 2024 were retrospectively examined. Demographic characteristics, metastasis type, metastasis locations, treatments applied, location of the primary tumor, histological type of the tumor, and metastasectomy status of the patients included in the study were evaluated. All patients underwent surgery for primary lung cancer. Generally, the mass in the lung was excised first. The metastasis was then removed. When brain surgery became a priority in some brain metastases, the metastasis was first removed and then the lesion in the lung was completely removed. In patients with oligometastasis, the tumor was either completely removed surgically or a complete cure was achieved with radiotherapy. All patients were stage 4a patients with metastases. The median age of the patients was 61 (36–76) years. 31 (52.6%) of the patients were aged 60 and over. 96.6% (n:57) of the patients were male and 3.4% (n:2) were female. Histopathological diagnosis was 35.6% squamous cell carcinoma (SCC) and 42.4% adeno cancer. 61.0% of the patients had brain metastases and 23.7% had adrenal metastases. The hospital stay of the patients was 14.0 ± 9.9 days. Disease-free survival time was 18.3 ± 24.4 months and overall survival time was 13.6 ± 11.5 months. While 32.2% (n:19) of the patients were alive, 67.8% (n:40) died. The survival rate was statistically significantly higher in patients who underwent metastasectomy compared to those who did not undergo metastasectomy (*p* = 0.027). The risk factors were found to be significantly associated with survival in the logistic regression analysis included metastasectomy (OR: 3.942, *p* = 0.030), diagnosis (SCC) (OR: 9,000, *p* = 0.042), recurrence (OR: 5.248, *p* = 0.012), adjuvant RT (OR: 0.298, *p* = 0.045), and neoadjuvant therapy (OR: 4.154, *p* = 0.040). In stage 4a lung cancer patients with oligometastasis, curative treatment of metastasis (metastasectomy) has a positive effect on survival. The low rate of radiotherapy and chemotherapy treatments given after metastasectomy will protect patients from the side effects of these treatments.

## Introduction

According to the World Health Organization (WHO) classification published in 2022, 90–95% of lung tumors are carcinomas, 5% are bronchial carcinoids, 2–5% are mesenchymal tumors and others [1]. The most common carcinomas are squamous cell carcinoma, adenocarcinoma, large cell carcinoma and small cell carcinoma [2]. Lung cancer ranks first among the cancers seen in the world and is the most common cause of cancer-related death [1]. Approximately 30% of cases with non-small cell lung cancer are limited to the lung and can only be cured with surgical treatment. While 30% of patients require multimodal treatment, 40% present with metastatic disease [3].

In tumor node metastasis (TNM) staging, the metastatic status of patients is defined as the absence of metastasis as M0 and the presence of metastasis as M1. In the 8th TNM staging, which was implemented in 2018, M1; It is divided into three subgroups: M1a, M1b and M1c. While M1a is unchanged, extrathoracic single-organ oligometastasis is classified as M1b and extrathoracic multiple-organ metastasis is classified as M1c [4]. The creation of the M1b group in the new staging is an indicator of the positive results of metastasis treatment on patient survival in oligometastatic diseases [4, 5]. Survival is poor in stage IV non-small cell lung cancer, but lung resection and complete resection or curative treatment of metastases are reported to contribute to survival in oligometastatic patients, which constitute a small portion of these patients [2, 6].

There are differences in the management of surgical and medical treatments in the treatment of oligometastatic lung cancer in studies in the literature [7–9]. It is important for survival to determine whether metastasectomy is beneficial or not and in what cases it should be performed.

The aim of our study is to evaluate the impact of metastasectomy on survival in patients with oligometastatic stage 4 lung cancer.

## Method

### Study design

This study was approved by the Ethics Committee. The Declaration of Helsinki protocol was followed in the research protocol. Our study is a retrospective study.

Cases with oligometastatic lung cancer operated on in our clinic between January 2015 and January 2024 were retrospectively examined.

Inclusion criteria for the study: Being diagnosed with lung cancer and having extrapulmonary solid organ metastasis.

Exclusion criteria from the study were: Surgery being inoperable (extrathoracic multiple organ metastasis is classified as M1c) and anatomic lung resection not being possible. While N2 + positivity was generally an exclusion criterion for surgical eligibility, exceptions were made for selected cases based on multidisciplinary team decisions considering clinical status and potential surgical benefits.

Demographic characteristics, metastasis type, metastasis locations, treatments applied, location of the primary tumor, histological type of the tumor, metastasectomy status, and survival outcomes in relation to lymph node involvement (N0, N1, N2, and N3) were evaluated.

All patients underwent surgery for primary lung cancer. Generally, the mass in the lung was excised first. The metastasis was then removed. In patients with brain metastases, metastasis treatment was performed with cranial surgery or stereotactic body radiation therapy (SBRT). Metastasis treatment was performed by the joint decision of neurosurgery and radiation oncology, according to the number of metastases, location and size of the metastasis. When brain surgery became a priority in some brain metastases, the metastasis was first removed and then the lesion in the lung was completely removed. Patients who were not suitable for metastasectomy were first given radiotherapy (RT) and then the primary mass was removed. Metastasectomy was performed after lung resection in patients with adrenal, splenic and ocular metastases. In patients with oligometastasis, the tumor was either completely removed surgically or complete cure was achieved with radiotherapy.

### Statistical analysis

IBM statistics SPSS v27 was used for patient records and statistical analysis. Descriptive statistics will be shown as mean ± standard deviation for variables with normal distribution, median (minimum–maximum) for variables with non-normal distribution, and number of people (n) and (%) for nominal variables. The Mann–Whitney test was preferred to compare the quantitative data between two groups not normally distributed. Student's T-test was utilized for comparison of the quantitative data between two normally distributed groups. Nominal variables will be evaluated with the pearson chi-square / Fisher Exact Test. Multivariable logistic regression analysis was performed to determine the factors affecting the dependent variable. Results for *p* < 0.05 were considered statistically significant.

## Results

The sociodemographic findings and disease information of the patients are shown in Table 1. The number of patients included in the study was 59. All patients were stage 4 patients with metastases. The median age of the patients was 61 (36–76) years. 31 (52.6%) of the patients were aged 60 and over. 96.6% (n:57) of the patients were male and 3.4% (n:2) were female. The median mass Standardized Uptake Values (SUV) value of the patients was 12.0 (1.2–32.6). The PET scan results indicate lymph node involvement among the patients as follows: 15 patients (25.4%) with N1 + , 14 patients (23.7%) with N2 + , and 4 patients (6.8%) with N3 + . The N0 + N1 group has a median disease-free survival of 9.0 months (range 0–120) and an overall survival time of 15.5 months (range 0–55). The N2 + N3 group has a median disease-free survival of 4.0 months (range 0–80) and an overall survival time of 3.0 months (range 0–18). In terms of location of the primary tumor, 21 (35.6%) were located in the upper right and 16 (27.1%) were located in the upper left. In terms of primary surgical site, right upper lobectomy was performed in 25.8%, left upper lobectomy in 19.0%, and left pneumonectomy in 19%. Lung resection was performed for the primary tumor an average of 24 days (17–48 days) after metastasectomy. Complications were observed in 17.0% (n:10) of the patients. Histopathological diagnosis was 35.6% squamous cell carcinoma (SCC) and 42.4% adeno cancer. 61.0% of the patients had brain metastases and 23.7% had adrenal metastases. Metastasectomy operation was performed in 39 (66.1%) of the patients. 61.0% of patients received radiotherapy (RT). Among the patients recommended for neo-adjuvant therapy, 10 (17.0%) patients accepted this treatment. Recurrence was observed in 55.9% of the patients. The hospital stay of the patients was 14.0 ± 9.9 days. Disease-free survival time was 8 (0–120) months and overall survival time was 12 (0–55) months. While 32.2% (*n*:19) of the patients were alive, 67.8% (n:40) died (Table 1).

**Table 1:** Sociodemographic findings and disease information of the patients

	*N* (%), Mean ± SD	Median (min–max)
Age (Years)	59.93 ± 8.9	61 (36–76)
Decade		
4. (30–39)	1 (1.7%)	
5. (40–49)	6 (10.2%)	
6. (50–59)	21 (35.6%)	
7. (60–69)	22 (37.3%)	
8. (70–79)	9 (15.3%)	
Gender		
Male	57 (96.6%)	
Female	2 (3.4%)	
Mass SUV (Standardized Uptake Values)	12.5 ± 5.6	12.0 (1.2–32.6)
Positron emission tomography (PET)		
N1 +	15 (25.4%)	
N2 +	14 (23.7%)	
N3 +	4 (6.8%)	
Location		
Upper right	21 (35.6%)	
Upper left	16 (27.1%)	
Others	22 (37.3%)	
Operation		
Right upper lobectomy	15 (25.8%)	
Left upper lobectomy	11 (19.0%)	
Left pneumonectomy	11 (19.0%)	
Others	22 (37.2%)	
Complication	10 (17.0%)	
Prolonged air leak	1	
Bronchopleural fistula (BPF)	2	
Respiratory failure	2	
Atelectasis	1	
Pneumonia	3	
Chylothorax	1	
Diagnosis		
Squamous cell carcinoma (SCC)	21 (35.6%)	
Adeno cancer	25 (42.4%)	
Others	13 (22.0%)	
Mass diameter (cm)	4.98 ± 2.4	
Site of metastasis		
Brain	36 (61.0%)	
Adrenal	14 (23.7%)	
Other	9 (15.3%)	
Metastasectomy		
No	20 (33.7%)	
Yes	39 (66.1%)	
Radiotherapy (RT)	36 (61.0%)	
Grade		
IVa	59 (100.0%)	
Neoadjuvant therapy	10 (17.0%)	
Adjuvant chemotherapy (CT)	48 (81.4%)	
Adjuvant RT	22 (37.3%)	
Relapse	33 (55.9%)	
Duration of hospital stay (day)	14.0 ± 9.9	11 (4–58)
Disease-free survival (month)	18.3 ± 24.4	8 (0–120)
Overall survival time (month)	13.6 ± 11.5	12 (0–55)
Survival		
Alive	19 (32.2%)	
Died	40 (67.8%)	

Comparison of patients who underwent and did not undergo metastasectomy is shown in Table 2. Although the hospital stays, disease-free survival time, and overall survival time were longer in patients who underwent metastasectomy compared to those who did not undergo metastasectomy, there was no statistical significance (*p* > 0.05). There was no statistical significance between the groups in terms of recurrence, adjuvant CT, adjuvant RT, and neoadjuvant therapy (*p* > 0.05). The survival rate was statistically significantly higher in patients who underwent metastasectomy compared to those who did not undergo metastasectomy (*p* = 0.027). The recurrence was seen 21 (53.8%) patients after metastasectomy. However, the recurrence rate, adjuvant CT, adjuvant RT, and neoadjuvant therapy rate were lower in patients who underwent metastasectomy. Hospital stays, overall survival time and disease-free survival time were longer in patients who underwent metastasectomy (Table 2). In brief, the quality of life in patients who underwent metastasectomy appears to be better in terms of overall survival and survival status. They tend to live longer and have higher survival rates, although there are no significant differences in disease-free survival, hospital stay duration, or relapse rates.

**Table 2:** Comparison of patients who underwent and did not undergo metastasectomy

	Metastasectomy		P* value
	No (N:20)	Yes (N:39)	
Age (years)			0.415
< 60	55.0%	43.6%	
> 60	45.0%	56.4%	
Site of metastasis			0.128
Brain	9 (45.0%)	27 (69.2%)	
Adrenal	7 (35.0%)	7 (17.9%)	
Other	4 (20.0%)	5 (12.8%)	
Duration of hospital stay (day)	15.0 ± 9.6	13.5 ± 10.2	0.604
Disease-free survival (month)	11.47 ± 7.2	14.96 ± 13.6	0.304
Overall survival time (month)	15.16 ± 20.6	19.9 ± 26.3	0.458
Survival			0.027
Alive	3 (15.0%)	16 (41.0%)	
Died	17 (85.0%)	23 (59.0%)	
Relapse	12 (60.0%)	21 (53.8%)	0.659
Adjuvant chemotherapy (CT)	17 (85.0%)	33 (84.7%)	0.815
Adjuvant radiotherapy (RT)	9 (45.0%)	13 (33.3%)	0.651
Neoadjuvant therapy	2 (10.0%)	8 (20.5%)	0.274

^*^Student’s t test

The effect of parameters in predicting survival is shown in Table 3. The risk factors were found to be significantly associated with survival in the logistic regression analysis included metastasectomy (OR: 3.942, *p* = 0.030), diagnosis (SCC) (OR: 9,000, *p* = 0.042), recurrence (OR: 5.248, *p* = 0.012), adjuvant RT (OR: 0.298, *p* = 0.045), and neoadjuvant therapy (OR: 4.154, *p* = 0.040) (Table 3). For patients with oligometastatic stage 4 lung cancer, undergoing surgical removal of metastases (metastasectomy) is likely to significantly improve survival outcomes. Additionally, avoiding recurrence, receiving specific therapies like adjuvant radiotherapy and neoadjuvant therapy, and understanding the type of cancer (e.g., squamous cell carcinoma) are important factors in managing their prognosis and overall survival.

**Table 3:** The effect of parameters in predicting survival

Variable	Odds ratio	95% CI	P* value
Metastasectomy	3.942	2.98–15.72	0.030
Radiotherapy (Yes)	1.143	0.37–3.52	0.816
Decadent (over 60 years old)	1.028	0.57–1.85	0.927
Diagnosis (Squamous cell carcinoma = SCC)	9.000	1.82–82.49	0.032
Site of metastasis (Brain)	0.606	0.29–1.24	0.173
Complication (No)	2.125	0.45–11.14	0.373
Recurrence (No)	5.248	2.76–15.64	0.012
Adjuvant chemotherapy (CT) (Yes)	1.122	0.29–4.22	0.865
Adjuvant radiotherapy (RT) (Yes)	0.487	0.08–1.00	0.045
Neoadjuvant therapy (Yes)	4.154	2.00–17.10	0.040

^*^Multiple logistic regression analysis

All patients underwent surgery for primary lung cancer. Additionally, survival comparisons were conducted for patients based on lymph node involvement. Patients with N0 and N1 lymph node status demonstrated significantly better survival outcomes compared to those with N2 and N3 involvement (*p* < 0.05) (Table 4.).

**Table 4:** Survival analysis by lymph node status

Lymph node status	Median disease-free survival (months)	Range (months)	Median overall survival (months)	*p*-value
N0 + N1	9.0	0–55	15.5	< 0.05
N2 + N3	4.0	0–18	3.0	< 0.05

Univariate and multivariate analyses to assess the impact of lymph node involvement on survival outcomes are shown in Table 5. Multivariate analysis revealed that N2 and N3 positivity were independent predictors of reduced survival (adjusted OR: 3.21; 95% CI 1.57–6.55; *p* = 0.002) (Table 5.).

**Table 5:** Univariate and multivariate analyses to assess the impact of lymph node involvement on survival outcomes

Variable	Univariate analysis (*p*-value)	Multivariate analysis (odds ratio)	95% Confidence interval (CI)	Multivariate analysis (*p*-value)
Lymph node involvement (N2 + N3)	< 0.05	3.21	1.57–6.55	0.002
Age > 60	0.087	1.85	0.87–3.92	0.091
Adjuvant radiotherapy	< 0.05	0.48	0.20–1.14	0.045
Neoadjuvant therapy	< 0.05	4.15	2.00–8.60	0.001

## Discussion

In this study, the effects of curative treatment of metastasis (metastasectomy) in stage 4 oligometastatic lung cancer, whether it is beneficial or not, and in what cases it should be applied were evaluated. In previous years, patients with stage IV lung cancer remained conservative and the survival of these patients was limited to 8–11 months. Survival in cases with oligometastatic disease is increasing with new surgical techniques, recent advances in staging, and large series published. In our study, the survival rate was higher in patients who underwent metastasectomy. However, the recurrence rate, adjuvant RT, and neoadjuvant treatment rates were lower in patients who underwent metastasectomy. In addition, hospital stay, overall survival time, and disease-free survival time were longer in patients who underwent metastasectomy.

In the literature, the average survival in patients with Stage IV lung cancer is reported to be 8–11 months, and the 5-year survival is below 1% [10]. In the seventh TNM staging system published by IASLC (International Association for the Study of Lung Cancer), Stage IV lung cancer includes the presence of distant metastases, independent of T and N [4]. In the eighth TNM staging, which was introduced in 2018, distant organ metastases are divided into single extrathoracic metastases M1b and multiple metastases in one or more organs M1c [4]. According to the metastasis status, Stage IV, M1a and M1b are divided into Stage IVa, and M1c is divided into Stage IVb [5]. Survival median for M1a = 11.5 months, median survival for M1b is 11.4 months, and median survival for M1c is 6.3 months [11]. In our study, all 59 patients were at Stage IVa. The average survival was 13.6 ± 11.5 months, which was higher than the literature.

The presence of metastases in cancer patients indicates that the disease is in the final stage and life expectancy is short [12]. However, in 1995, Hellman and Weichsellbaum declared that oligometastatic diseases were a special subgroup and showed that local treatment could be curative in this group, leading to a shift towards aggressive treatment in advanced-stage disease [13]. Oligometastasis refers to solitary metastasis with limited organ involvement and low metastatic burden [7]. While the definition of oligometastasis used to refer to a single organ and a single metastasis, it has changed over time to 1–3 metastases in a single organ [14]. Although the incidence of oligometastatic non-small cell lung cancer is reported to be between 7 and 25%, the actual prevalence is not exactly known [15, 16].

Studies in the literature recommend PET/CT evaluation and invasive mediastinal staging for extrapulmonary metastasis in candidates with isolated brain metastases and curative treatment is planned. In the presence of N0/1 synchronous primary lung cancer and isolated brain metastasis, surgical resection or radiosurgery ablation is recommended for isolated brain metastases if there is no other metastasis [7, 12, 17]. In our study, in line with the literature, the brain metastasis was generally excised first. The lung mass was then removed. Metastasis treatment was performed by cranial surgery or stereotactic body radiation therapy (SBRT) in 36 (61.0%) cases with brain metastases. In 9 (45.0%) patients who were not suitable for metastasectomy, radiotherapy (RT) was first given and then the primary mass was removed. Lung resection was performed for the primary tumor an average of 24 days (17–48 days) after metastasectomy. Metastasectomy was performed after lung resection in 12 (30.7%) patients with adrenal, splenic and ocular metastases. In patients with oligometastasis, the tumor was either completely removed surgically or a complete cure was achieved with radiotherapy.

When tumor histology was examined in our series, it was found that 42.4% (*n* = 25) of the cases had adenocarcinoma and 35.2% (*n* = 21) had squamous cell carcinoma. Consistent with the literature, adenocarcinoma was the most common histological type [14, 18]. In the survival analysis by cell type in Plones et al.'s study, the median survival was 18.1 months in adenocarcinoma histology and 14.1 months in squamous cell carcinoma histology, and no significant difference was found in the survival analysis by histological type [19]. In our study, median survival in survival analysis by cell type was 14.93 months in adenocarcinoma histology and 10.27 months in squamous cell carcinoma histology, and there was no significant difference in survival analysis by histological type (*p* = 0.195). In the reviews of Novoa et al. and Ashwort et al., adenocarcinoma subtype was reported to be a good prognostic factor [20, 21]. In the series of Kabalak et al. and Fernandez et al., there was no difference in prognosis when adenocarcinoma histology was compared with others [7, 12]. In our study, when cell types were compared, there was no statistical difference in terms of survival between groups (*p* > 0.05).

In lung cancer, while extrathoracic metastases are most commonly observed in the brain and adrenal gland, metastatic disease can also be seen in bone, liver, spleen, kidney and lymph nodes. It is known that the most common extrathoracic metastasis in patients with non-small cell lung cancer is brain metastasis [22–24]. Brain metastases are present in 25–35% of patients with stage IV lung cancer, and survival is 1–2 months in untreated cases, 2 months with steroid treatment alone, and 3–6 months in those treated with WBRT (whole brain radiotherapy) [25, 26]. In Fleckenstein et al.'s series of 75 cases, overall survival was given as 21.3 months [9]. Xu et al. reported that overall survival after synchronous brain metastasis was 12.3 months, median survival was 15.4 months in patients who underwent complete surgical resection, and 11.5 months in patients who underwent WBRT (whole brain radiotherapy) [27]. Bougie et al., in their series of 115 cases with isolated brain metastases, reported overall survival as 7.8 months in the stereotactic radiosurgery (SRS) group and 13.3 months in the surgery group [28]. In none of these studies was metastasis treatment choice associated with survival [9, 25–28]. Contrary to studies in the literature, in our study, 36 (61.0%) of 59 patients with Stage IV lung cancer had brain metastases. The overall survival time was 13.6 months, which was longer than the literature. However, the survival time in patients with brain metastases who underwent metastasectomy was 19.9 months. Therefore, we can say that metastasectomy in brain metastases prolongs survival time.

In cases with synchronous brain metastasis, the operating strategy should be to treat the metastasis first [29]. Metastasis treatment can be done with cranial surgery, radiosurgery, WBRT (whole brain radiotherapy) or post-surgical radiosurgery [8, 30]. Although studies in the literature do not make a specific recommendation for curative resection, WBRT is recommended after curative resection [7, 29]. In our study, 20.8% (*n* = 9) of 36 patients with synchronous isolated brain metastases underwent stereotactic body radiation therapy (SBRT), and 69.2% (*n* = 27) underwent cranial surgery and metastasectomy. There was no statistical difference in terms of metastasis and primary tumor treatment. In the postoperative period, adjuvant CT was applied to 29 (80.6%) of the cases with brain metastases, adjuvant RT was applied to 15 (41.7%), and neoadjuvant treatment was applied to 5 (13.9%). When the applied treatments were compared, there was no statistically significant difference in survival (*p* > 0.05). However, adjuvant RT and neoadjuvant therapy had statistically significant effects on survival. Additionally, recurrence was observed in 21 (53.8%) patients after metastasectomy and was less than in those without metastasectomy. However, the low rate of metastasectomy and recurrence had statistically significant effects on survival.

The inclusion of N2 + and N3 + patients in this study introduces both challenges and opportunities for interpretation. While N2 positivity is often considered an exclusion criterion for surgical resection in metastatic lung cancer due to its association with poor prognosis, selected cases in our study were included based on multidisciplinary team decisions, weighing potential benefits against risks. This inclusion reflects real-world clinical scenarios but may impact the generalizability of the results. The findings suggest that carefully selected patients with N2 or N3 involvement can benefit from surgical intervention, as evidenced by improved survival in some cases. However, these results should be interpreted cautiously and supported by larger studies. The heterogeneity of this subgroup highlights the importance of individualized treatment approaches and suggests that lymph node status should remain a critical factor in patient selection for surgical interventions [6, 9, 16].

In metastatic lung cancer management, the sequence of metastasis and primary tumor resection plays a critical role in optimizing patient outcomes. Current literature supports treating brain metastases before lung resection to minimize risks associated with general anesthesia and to prevent immunosuppression-related progression of brain disease [25, 27]. Adrenal metastases, on the other hand, can often be resected synchronously with lung surgery [16]. For bone metastases that do not require surgical stabilization, postoperative radiotherapy is a preferred approach [18, 29]. Tailoring the sequence of treatments based on the type and location of metastases is essential for improving survival and minimizing complications. Our study primarily followed these principles, with the treatment strategy being determined collaboratively by a multidisciplinary team based on individual patient characteristics.

There are some limitations in our study. The retrospective design, single-center setting, and small sample size limit the generalizability and comprehensiveness of our findings. Another limitation of this study is the lack of comprehensive data on next-generation sequencing (NGS) and PD-L1 expression levels, which have become standard diagnostic tools in the era of immunotherapy and targeted therapies. While we included a limited number of N2 + and N3 + patients based on multidisciplinary team decisions, this deviation from standard selection criteria reflects real-world practice but may introduce heterogeneity into the data. Additionally, not all patient data could be accessed from hospital records. Information such as treatment-related side effects, treatment doses, body surface area, height, weight, and KRAS, NRAS, and BRAF mutation results were unavailable. The limited availability of data, especially regarding neoadjuvant therapy and its rationale for use or omission, constrains the depth of the analysis.

## Conclusion

We found that curative treatment of metastasis (metastasectomy) has a positive effect on survival in stage 4 lung cancer patients with oligometastasis. In selected groups of oligometastatic patients, the survival of patients can be extended with complete resection of the primary tumor and curative treatment of metastasis. In our study, although cases with brain metastasis had worse survival compared to other metastatic cases, we see that survival increases with aggressive treatment in lung cancer cases with oligometastasis, in line with current data.

In conclusion, the quality of life in patients who underwent metastasectomy appears to be better in terms of overall survival and survival status. The patients tend to live longer and have higher survival rates, although there are no significant differences in disease-free survival, hospital stay duration, or relapse rates. The significant difference in adjuvant chemotherapy use suggests that postoperative treatment might also play a role in these outcomes. We also think that the low rate of radiotherapy and chemotherapy treatments given after metastasectomy will protect patients from the side effects of these treatments. Our series and increasing studies in the literature show that survival increases in cases treated with bifocal surgery. Large series and further studies are needed to determine which patient subgroups will benefit most from this type of bifocal surgery.

## Data Availability

The availability of data and materials: The datasets used and/or analyzed in this study are available from the author upon reasonable request.
